# Lipid-lowering statins and polyphenol-based supplementation: a scoping review on drug-food interaction potential

**DOI:** 10.3389/fphar.2025.1541871

**Published:** 2025-05-30

**Authors:** Rita Costa, Carolina Ferreira, André Alves, Sara Nunes, Flávio Reis, João Malva, Sofia Viana

**Affiliations:** ^1^ Polytechnic University of Coimbra, Coimbra, Portugal; ^2^ Faculty of Medicine, Institute of Pharmacology and Experimental Therapeutics, University of Coimbra, Coimbra, Portugal; ^3^ Coimbra Health School, H&TRC- Health and Technology Research Center, Polytechnic University of Coimbra, Coimbra, Portugal; ^4^ Coimbra Institute for Clinical and Biomedical Research (iCBR) - Center for Innovative Biomedicine and Biotechnology (CIBB), University of Coimbra, Coimbra, Portugal; ^5^ Faculty of Medicine, Clinical Academic Center of Coimbra (CACC), University of Coimbra, Coimbra, Portugal

**Keywords:** statins, polyphenols, phytochemicals, drug-food/herb interactions, biologic mechanisms, pharmacokinetics, pharmacodynamics

## Abstract

**Background:**

Lifestyle modifications, particularly the adoption of healthy dietary patterns such as the Mediterranean Diet (MedDiet), are foundational in any treatment plan, including for patients prescribed first-line statin therapy for hypercholesterolemia. However, the rising popularity of MedDiet-associated foods and nutraceuticals among health-conscious consumers has raised concerns about their potential interactions with statins, potentially leading to adverse effects. One notable example involves polyphenol supplements, a class of anti-dyslipidemic phytochemicals known to influence statins’ pharmacokinetics. Still, whether chronic polyphenol exposure achieves plasma concentrations sufficient to alter statin pharmacokinetics in clinical settings is controverse. Moreover, it remains unclear which key biological targets are shared by both classes of molecules and how they mediate potential pharmacokinetic and pharmacodynamic interactions. This study aims to systematically map reported statin-polyphenol interactions and identify the principal biological targets involved, elucidating their impact on statin pharmacokinetics, efficacy, and toxicity.

**Methods:**

A scoping review was conducted using the PubMed/Medline, Scopus, and Web of Science databases. This work was designed in accordance with PRISMA-ScR. The review protocol was registered in the Open Science Framework.

**Results:**

Statin-polyphenol interactions were reported in 83.9% of the studies analyzed. The biological targets mediating these interactions play chief roles in statins’ cellular uptake (OATP/P-glycoprotein), metabolism (CYP450/intestinal esterases), and core mechanisms underlying statin action, namely, HMG-CoA reductase inhibition. Polyphenols significantly influenced statin pharmacokinetics, altering total drug exposure (AUC) and extent (Cmax), either by enhancement or inhibition, with apparently less impact on statins’ absorption rate (Tmax). At the clinical level, only five studies addressed statin-polyphenol interactions, yielding conflicting results on the potential enhancement of therapeutic efficacy and adverse effects.

**Discussion:**

This work presents an integrated perspective on the cellular and molecular mechanisms underlying statin–polyphenol interactions. It highlights substantial inter-individual variability linked to conflicting evidence, from synergistic effects enabling lower statin doses and reduced adverse events to potential exacerbation of side effects. These findings underscore the need for controlled studies to clarify the clinical relevance of polyphenol-induced alterations in statin pharmacokinetics and pharmacodynamics. Such data are essential to develop evidence-based guidelines that may optimize statin therapy and support individualized treatment strategies.

**Systematic review registration:**

identifier 10.17605/OSF.IO/DJW5G.

## 1 Introduction

Statins are the first-line treatment for hypercholesterolemia and play a key role in the prevention of cardiovascular diseases (Loeffen et al., 2015). They are selective, competitive inhibitors of the rate limiting enzyme 3-hydroxy-3-methylglutaryl-coenzyme A (HMG-CoA) reductase responsible for converting HMG-CoA to mevalonate in the cholesterol synthesis pathway (Attardo et al., 2022). By decreasing cholesterol synthesis, hepatic low-density lipoproteins (LDL) receptors are induced through a feedback loop leading to increased LDL-cholesterol uptake from the circulation. This results in reduced serum levels of LDL and triglycerides while increasing high-density lipoprotein (HDL) concentrations (Sirtori, 2014). Statins possess distinct pharmacokinetic profiles that are intrinsically tied to their physicochemical properties. Simvastatin and lovastatin, administered as prodrugs containing a lactone ring, exhibit higher lipophilicity compared to statins with open acid structures (Schachter, 2005; Shitara and Sugiyama, 2006). All statins exhibit very low systemic bioavailability due to an extensive first-pass effect at the intestinal and/or hepatic level, with the notable exception of pitavastatin. This characteristic can be advantageous, as the liver - the primary site of cholesterol biosynthesis - is the target organ for statins (García et al., 2003).

For lipophilic statins (e.g., fluvastatin, atorvastatin, lovastatin, simvastatin, pitavastatin), passive diffusion across hepatocyte cell membranes is a key mechanism for efficient first-pass (Hamelin and Turgeon, 1998). Conversely, hydrophilic statins, such as rosuvastatin and pravastatin, rely primarily on active carrier-mediated transport mainly through the organic anion-transporting polypeptides 1 (OATP1B1, also referred to as SLCO1B1, OATPC/LST-1, or SLC21A6), which confers greater hepatoselectivity (Hamelin and Turgeon, 1998; Attardo et al., 2022). Notably, certain lipophilic statins, including cerivastatin, pitavastatin, and atorvastatin, are also recognized as substrates of OATP1B1 (Sirtori, 1993; Hsiang et al., 1999; Shitara and Sugiyama, 2006). Due to their extensive intestinal and hepatic metabolism primarily through CYP450, statins are minimally excreted in unchanged form via the kidneys and are primarily excreted in bile and feces through specific efflux transporters such as the multidrug resistance-associated protein 2 (ABCC2), multidrug resistance 1 (ABCB1), breast cancer resistance protein (ABCG2) as well as P-glycoprotein (P-gp), also known as multidrug resistance protein 1 (MDR1) (Wessler et al., 2013; Wiggins et al., 2016).

Although statins are widely considered safe medications, they can cause dose-dependent adverse effects, including hepatic and muscle-related complications, which are more likely to occur at elevated plasma concentrations (Mollazadeh et al., 2021). Notably, statin absorption is significantly influenced by the timing of administration and concurrent food intake, as food can alter their pharmacokinetics or pharmacodynamics, potentially increasing the risk of adverse reactions or diminishing their pharmacological efficacy. For instance, lovastatin is absorbed more effectively when taken with food (Garnett, 1995), whereas the absorption of fluvastatin, atorvastatin, and pravastatin is reduced under similar conditions (Pan et al., 1993; Smith et al., 1993; Radulovic et al., 1995). Beyond the specific statin, the type of food components consumed also plays a pivotal role in determining the outcomes of drug-food or drug-herb interactions, with polyphenols serving as a prime example.

Polyphenols are abundantly found in a wide range of foods associated with the Mediterranean dietary pattern (Castro-Barquero et al., 2018), contributing to their sensory and nutritional qualities, such as astringency, aroma, and color (Issaoui et al., 2020; Bertelli et al., 2021). These compounds are plant secondary metabolites characterized by a phenolic ring as their basic structural unit and can be categorized into at least ten distinct classes based on their core chemical structure. Among these, flavonoids represent approximately two-thirds of the total dietary polyphenol intake, while phenolic acids account for the remaining one-third (Abbas et al., 2017). Flavonoids and other phenolic compounds are ubiquitous in nature and constitute the largest group of phytochemicals with antioxidant properties (Kumar and Goel, 2019). Consequently, extensive research has explored their beneficial effects as effective anti-inflammatory, anticancer, immunomodulators, prebiotics, cardioprotective and anti-dyslipidemic agents as they regulate key metabolic processes, including adipogenesis, lipolysis, fatty acid β-oxidation, AMPK/mTOR balance, to name a few (Aloo et al., 2023; Ferreira et al., 2024).

Only 5%–10% of total ingested polyphenols are absorbed in the small intestine, while the remaining 90%–95%, comprising unabsorbed compounds or those refluxed into the intestinal lumen, proceed to the colonic sections where they are metabolized by resident microbiota (Wang et al., 2022). Low molecular weight phenolic compounds, such as phenolic acids, are absorbed either through passive diffusion or via specific transporters located on enterocyte membranes, including P-glycoprotein (P-gp), sodium-glucose cotransporters (SGLT1), and OATPB1. At the metabolic level, polyphenols have been shown to inhibit CYP450 enzymes. Specifically, several flavonoids, including naringenin, biochanin A, genistein, epigallocatechin gallate (EGCG), and quercetin, have been identified as potent inhibitors of CYP3A4 and CYP2C9 isoforms (Le Goff et al., 2002; Satoh et al., 2016; Elbarbry et al., 2018). Moreover, biochanin A, quercetin and EGCG have exhibited the capacity to suppress hepatic HMG-CoA reductase activity, mirroring statins’ mechanism of action (Cuccioloni et al., 2011). Considering that high-dose polyphenol supplements are readily available over the counter for managing dyslipidemia and cardiovascular diseases (Setchell and Cassidy, 1999; Sung et al., 2004), the potential for clinically significant statin-polyphenol interactions is substantial.

Alterations in statin pharmacokinetics due to polyphenol co-administration have been implicated in statin-induced myopathy through the modulation of OATPs, CYP450 and HMG-CoA reductase activities (Zechner et al., 2022). Still, other candidate biological targets may also mediate statin-polyphenol interactions, potentially influencing both pharmacokinetic and pharmacodynamic responses and ultimately impacting the effectiveness of statins lipid-lowering therapies. Moreover, it remains unclear whether chronic consumption of polyphenol-based supplements can consistently achieve plasma concentrations sufficient to meaningfully alter statin pharmacokinetics in the clinic. This scoping review aims to identify the key biological targets that govern statin-polyphenol interactions to establish a novel mechanistic framework for a deeper understanding of their molecular basis. By doing so, it seeks to optimize the effectiveness of statin therapy, ultimately contributing to improved management of cardiovascular and metabolic diseases.

## 2 Material and methods

### 2.1 Study protocol

This scoping review was designed in accordance with the Preferred Reporting Items for Systematic Reviews and Meta-Analyses Extension for Scoping Reviews (PRISMA-ScR) and guided by the updated PRISMA 2020 checklist (Page et al., 2021a; Page et al., 2021b). The review protocol was registered in the Open Science Framework on September 6, 2024 (Viana and Galinha, 2024). The research question, eligibility criteria and search strategy were designed using the Participants-Concept-Context (PCC) framework. This scoping review focused on food-drug and/or herbal medicines-drug interactions (concept) between polyphenol-based supplementation and lipid-lowering statin drugs (context). Study population included original articles comprising *in vitro* models, *in vivo* models and human individuals (participants). The research question was: “What are the characteristics of reported interactions between statins and polyphenols in terms of pharmacokinetic and pharmacodynamic outcomes?” Based on this, the study had two main objectives: (1) to map the biological entities involved in statin-polyphenol interactions and (2) to characterize the associated pharmacokinetic outcomes. Only peer-reviewed, english-language *in vitro* and *in vivo* experimental studies were included, focusing on the primary biological targets mediating polyphenol-statin interactions and/or their pharmacokinetic effects. Exclusion criteria encompassed non-original research, such as book chapters, opinion pieces, narrative or systematic reviews, editorials, conference and meeting abstracts, errata, and study protocols. Additionally, studies lacking an abstract or inaccessible full texts were excluded. No timeframe restrictions were applied, with the latest search update dating back to 1993.

### 2.2 Search strategy

The literature search was conducted in the PubMed, Scopus, Web of Science databases. Search strategy was designed using keywords such as “Polyphenols”, “Statins”, “Interactions”, combined with Boolean operators and the following syntax: ((Polyphenols OR Flavonoids) AND (Statins) AND (Interactions)). The search profile will include text terms in title, abstract, Medical Subject Headings (MeSH) terms and subheadings. A customized data-extraction form was constructed and integrated into the Covidence software (Covidence, 2025), and duplicate entries were removed. Abstracts of the resulting articles were screened for eligibility. Subsequently, the full text of each article that met the inclusion criteria was manually searched for additional relevant publications. Titles and abstracts identified by the search were independently screened in duplicate by two independent reviewers against the eligibility criteria. Disagreements between the two reviewers during the screening and data extraction process were discussed and resolved by a third reviewer. Reasons for exclusion of full-text studies that do not meet the inclusion criteria were recorded and reported in the scoping. Articles that passed the initial screening were subjected a full-text review based on the inclusion criteria detailed above. Any disagreements that arise between the reviewers at each stage of the study selection process were resolved through discussion, or with a third reviewer.

### 2.3 Data items and synthesis of results

The extracted data included the following elements: 1) Study type, 2) Statin type/dose, 3) Polyphenol type/dose, 4) Occurrence of statin-polyphenol interactions, 5) Key biological targets, and 6) Pharmacokinetic outcomes. The results from all included studies were summarized and presented descriptively in dedicated evidence tables, complemented by bar and pie chart visualizations.

## 3 Results

### 3.1 Characterization of the included studies

**FIGURE 1:**
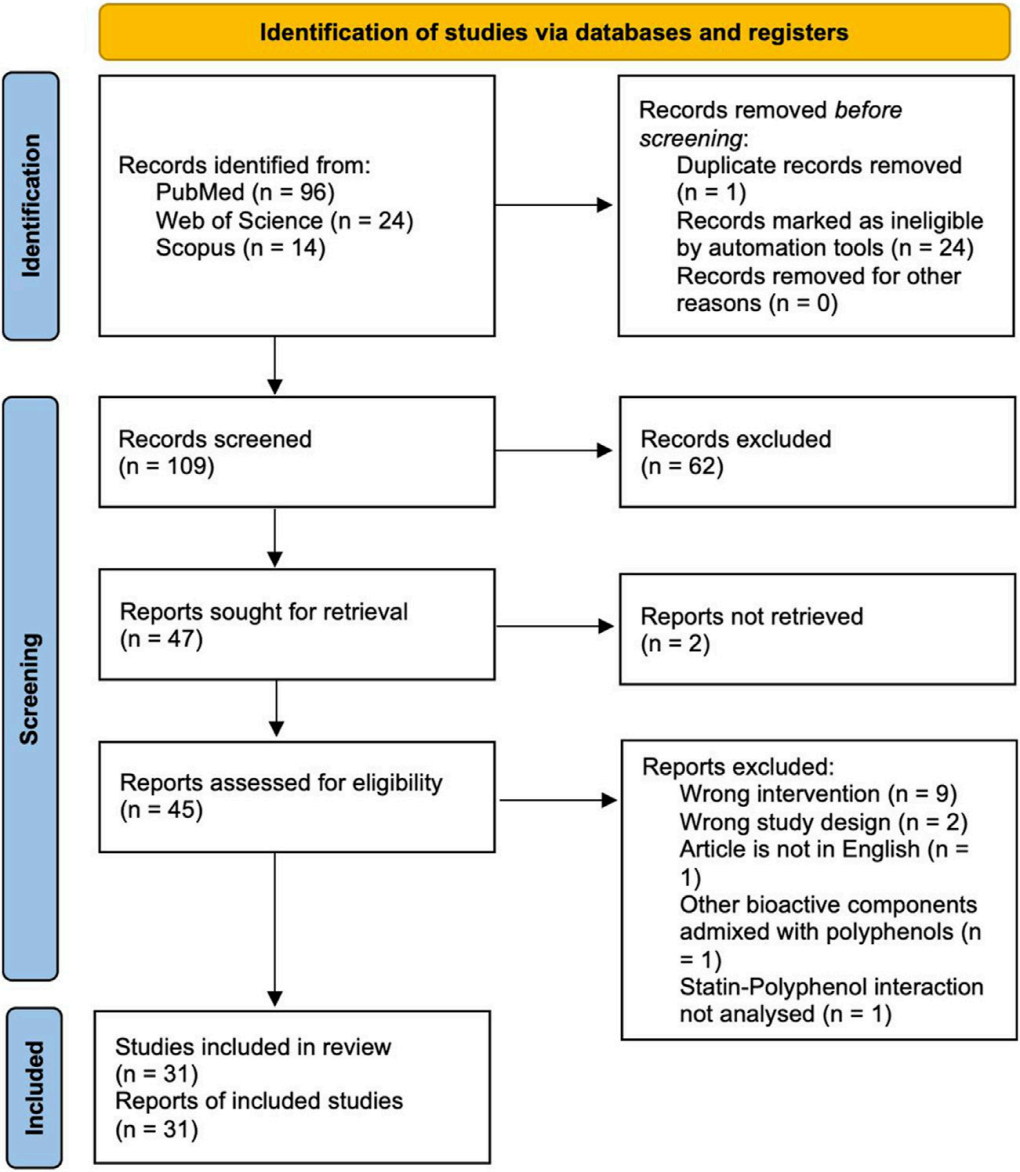
PRISMA 2020 flow diagram of study selection process.

The primary search identified 134 published papers, of which 96 were from PubMed, 24 from Web of Science and 14 from Scopus. A total of 25 duplicates were excluded, leaving 109 articles for screening. After thorough analysis, 78 articles were excluded for not meeting the inclusion criteria, leaving 31 eligible for full-text scrutiny. The results of the study selection process are shown in a PRISMA 2020 flow diagram (Figure 1).

**FIGURE 2:**
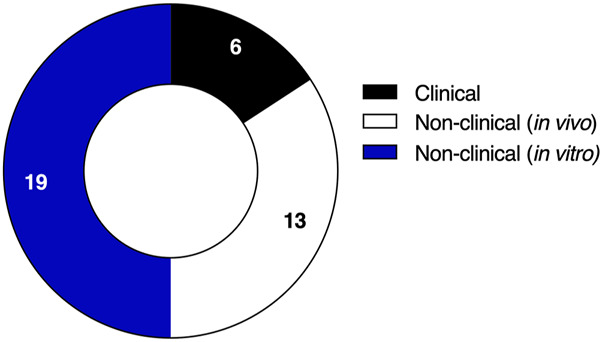
Type of studies enrolled in the included studies.

**FIGURE 3:**
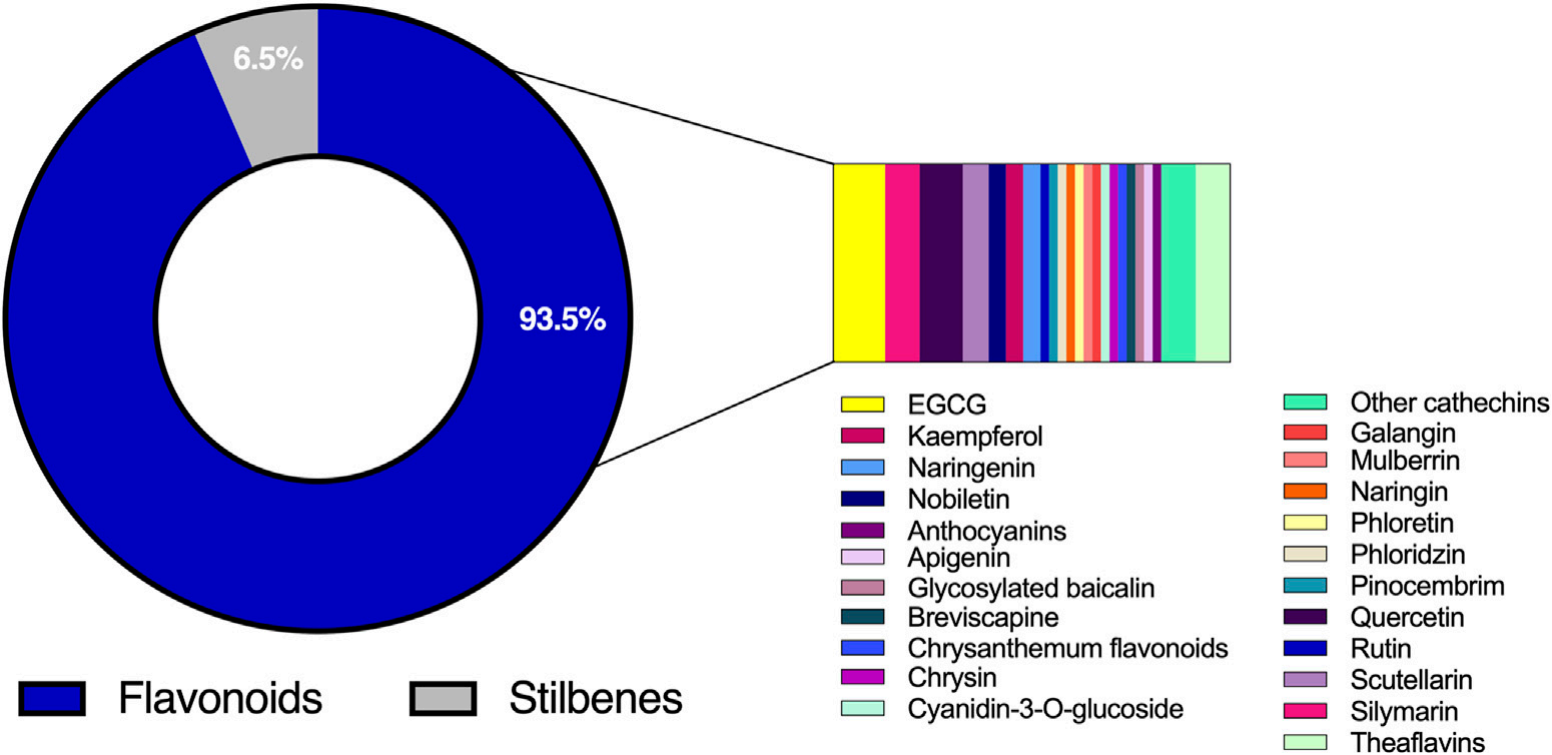
Proportion of studies reporting statin-polyphenol interactions.

The experimental and quasi-experimental studies that met the inclusion criteria were categorized into three major types: non-clinical (*in vitro and in vivo*) and clinical studies. Information collected were based on 19 *in vitro* studies, 13 *in vivo* studies and six clinical studies (Figure 2). Dataset covers seven distinct statins, including both pro-drugs and active forms. Rosuvastatin was the most investigated, appearing in 11 studies, followed by simvastatin in 10 studies, atorvastatin in 6, fluvastatin and pitavastatin each in 2, and lovastatin and pravastatin in one study each. With respect to polyphenols, most studies (93.5%, n = 29) concentrated on flavonoids, while only 6.5% (n = 2) focused on a single stilbene, resveratrol (Figure 3).

**FIGURE 4:**
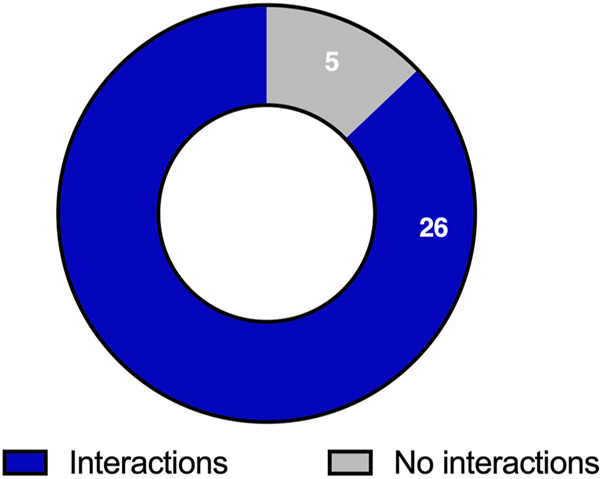
Proportion of studies reporting the biological targets mediating statin-polyphenol interactions.

Out of the 31 studies analyzed, 26 (83.9%) reported significant statin-polyphenol interactions, while 5 (16.1%) did not (Figure 4).

### 3.2 Statin-polyphenol interactions: main biological targets

Non-clinical studies (*in vitro*) have identified OATPs inhibition is the most reported biological target for statin-polyphenol interactions (11 studies), followed by inhibition of CYP enzymes (5 studies), P-gp (3 studies), HMG-CoA reductase (3 studies) and intestinal esterases (1 study).

A diverse array of polyphenols has been identified as inhibitors of OATPs-mediated statin uptake at both the hepatic and intestinal levels. For instance, silymarin, scutellarin, and glycosylated baicalein were shown to inhibit the hepatic OATP1B1 transport of statins when co-administered with rosuvastatin (Table 1, Fan et al. (2008), Kock et al. (2013), Liu et al. (2020b)). This effect was also observed for simvastatin, atorvastatin, and pravastatin when co-treated with flavonoids such as epigallocatechin-3-gallate (EGCG), apigenin, kaempferol, and quercetin (Mandery et al., 2012; Wu et al., 2012; Kim et al., 2017). Furthermore, inhibition of OATP1B3-mediated transport of atorvastatin, simvastatin, and rosuvastatin) was observed upon co-administration of flavonoids, such as EGCG, silymarin, scutellarin, apigenin, kaempferol, quercetin, naringenin, naringin, and rutin (Kock et al., 2013). At the intestinal level, theaflavins from black tea extracts were found to inhibit OATP2B1-mediated transport of rosuvastatin (Kondo et al., 2019) while EGCG and phloretin inhibited rosuvastatin uptake through OATP1A2 (Takahashi et al., 2021). Although less common, polyphenols were found to inhibit P-gp-mediated transport of statins as well (3 studies). This is the case of naringin and silymarin who inhibited the efflux of rosuvastatin and pitavastatin, respectively (Deng et al., 2008; Shirasaka et al., 2011a; Shirasaka et al., 2011b).

**TABLE 1:** Statin-polyphenol interactions: Main biological targets.

Statin [Dose]	Polyphenol [Dose]	Type of study	Main biological targets					References
			Inhibition of OATP-mediated uptake	Inhibition of P-gp efflux transporter	Inhibition of CYPs activity	Inhibition of HMG-CoAR	Inhibition of intestinal esterases	
Rosuvastatin [0.5 µM]	Chrysin, galangin, and pinocembrim [0–500 µM]	Non-clinical (*In vitro*)	X	-	-	-	-	Navratilova et al. (2018)
Rosuvastatin [0.01 µM]	Phloretin [10 µM] Phloridzin [100 µM]	Non-clinical (*In vitro*)	X	-	-	-	-	Takahashi et al. (2021)
Rosuvastatin [50 µM]	Scutellarin [50 µM]	Non-clinical (*In vitro*)	X	-	-	-	-	Liu et al. (2020a)
Rosuvastatin [0.5 μM]	Silymarin [100 µM]	Non-clinical (*In vitro*)	X	-	-	-	-	Kock et al. (2013)
Rosuvastatin [0.1 or 10 μM]	Silymarin [0.5–50 μM]	Non-clinical (*In vitro*)	X	X	-	-	-	Deng et al. (2008)
Simvastatin [0.5–1 μM]	EGCG [1–1,000 μM]	Non-clinical (*In vitro*)	X	-	X	-	-	Yang et al. (2017)
Simvastatin [50, 100 µM]	Naringenin [10, 25, 50 µM]	Non-clinical (*In vitro*)	-	-	X	-	-	Ubeaud et al. (1999)
Simvastatin [20, 50 or 100 μmol/L]	Naringenin [100, 150, or 300 μmol/L]	Non-clinical (*In vitro*)	-	-	X	-	-	Motawi et al. (2014)
Simvastatin [10–250 mM]	Bergamottin [10–100 µM]	Non-clinical (*In vitro*)	-	-	X	-	-	Le Goff-Klein et al. (2003)
Simvastatin [10 µM]	Resveratrol [30–100 mM]	Non-clinical (*In vitro*)	-	-	-	X	-	Wong et al. (2011)
Simvastatin [0.1 µM]	Resveratrol [30 µM]	Non-clinical (*In vitro*)	-	-	-	X	-	Villanueva et al. (2013)
Atorvastatin [0.5, 1.0, 2.5 µM]	Apigenin [10, 25, 50 µM] Kaempferol [10, 25, 50 µM] Quercetin [10, 25, 50 µM]	Non-clinical (*In vitro*)	X	-	-	-	-	Mandery et al. (2012)
Atorvastatin [4 µM]	Epicatechin-3-gallate, epigallocatechin, epicatechin, and gallocatechin gallate [40 µM]	Non-clinical (*In vitro*)	-	-	-	X	-	Lu et al. (2008)
Atorvastatin [100 µM] Fluvastatin [100 µM] Rosuvastatin [100 µM]	Chlorogenic acid [100 µM] Chrysanthemum flavonoids [50 μg/mL] Glycyrrhetinic acid [100 µM] Mulberrin [50 μg/mL] Quercetin [100 µM] Scutellarin [100 µM]	Non-clinical (*In vitro*)	X	-	-	-	-	Wen et al. (2016)
Lovastatin [5 µM]	Kaempferol [100 µM] Narigenin [100 µM]	Non-clinical (*In vitro*)	-	-	-	-	X	Li et al. (2007)
Fluvastatin [50 mM]	EGCG [0–100 µM]	Non-clinical (*In vitro*)	-	-	X	-	-	Misaka et al. (2018)
Pitavastatin [1 µM]	Naringin [1,000 µM]	Non-clinical (*In vitro*)	X	X	-	-	-	Shirasaka et al. (2011b)
Pravastatin [50 µM]	Quercetin [0–1,000 µM]	Non-clinical (*In vitro*)	X	-	-	-	-	Wu et al. (2012)
Pitavastatin [1 µM]	Naringin [1,000 µM] Naringin [200 or 1,000 µM]	Non-clinical (*In vitro*)	X	X	-	-	-	Shirasaka et al. (2011a)

Abreviations: EGCG, Epigallocatechin-3-gallate; HMG-CoAR, Hydroxymethylglutaryl-CoA (HMG-CoA) reductase; OATP, Organic Anion-Transporting Polypeptide; P-gp, P-glycoprotein SD, single dose; PO, *per os*; -, not determined.

Statin-polyphenol interactions were also found to be mediated by CYP450 enzymes (reported in five studies), particularly the CYP3A4 isoform, a key enzyme in statin metabolism. Polyphenols such as baicalein, breviscapine, and EGCG inhibited CYP3A4 activity upon statin co-administration (Lu et al., 2008; Ju et al., 2017; Meng et al., 2021). Breviscapine also reduced the expression of CYP3A4 mRNA, suggesting that the polyphenol affects both the enzyme’s activity and its synthesis at the transcriptional level, potentially exacerbating the risk of adverse reactions upon statin co-administration. Polyphenols not only inhibit CYP450 enzymes but also exhibit the capability to inhibit esterase activity in the intestine, thereby preventing the rapid degradation of statins. This is the case of kaempferol and naringenin who increased lovastatin cellular permeability due to their esterase-inhibitory effects (Li et al., 2007).

Finally, a common biological target of statin-polyphenol interactions was identified as HMG-CoA reductase, the enzyme targeted by statins to reduce cholesterol synthesis (reported in three studies). Resveratrol was found to potentiate the inhibitory effects of simvastatin on cholesterol biosynthesis and HMGCR enzyme activity and abolished the counter-regulatory stimulatory effects of simvastatin on HMGCR mRNA transcripts and protein expression in human endometrial stroma cells (Villanueva et al., 2013) and rat theca-interstitial cells (Wong et al., 2011). This evidence suggest that polyphenols may enhance the therapeutic benefits of statins by targeting multiple steps of cholesterol biosynthesis.

### 3.3 Statin-polyphenol interactions: pharmacokinetic outcomes

**FIGURE 5:**
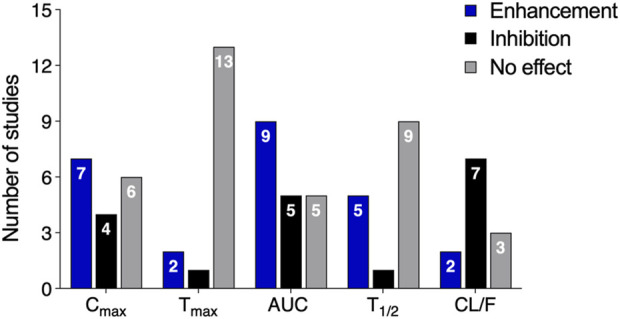
Descriptive analysis of statins’ pharmacokinetic parameters upon polyphenol co-administration (AUC, area under the plasma concentration-time curve; t1/2, half-life; CL/F, clearance adjusted for bioavailability).

The range of biological mechanisms through which statins may interact with polyphenols is particularly relevant to drug pharmacokinetics. Nevertheless, studies investigating the pharmacokinetic behavior of statins in the presence of polyphenol co-treatment have reported a wide array of results, as summarized in Figure 5.

In a comprehensive global analysis, non-clinical (*in vivo*) and clinical studies have revealed that polyphenols can significantly influence the pharmacokinetics of rosuvastatin, simvastatin, atorvastatin, and lovastatin, altering drug exposure either by enhancement or inhibition (Table 2). Specifically, fourteen studies reported changes in total statin exposure (AUC), with nine documenting an increase in AUC and five reporting a decrease, highlighting substantial variability in the magnitude of these effects. For instance, scutellarin and silymarin were shown to increase the extent of exposure (Cmax) of rosuvastatin and atorvastatin, respectively, while theaflavin decreased it (Malekinejad et al., 2014; Liu et al., 2020b). Notably, numerous polyphenols increased the exposure of statin pro-drugs: baicalein, breviscapine, and EGCG enhanced simvastatin Cmax (Ju et al., 2017; Yang et al., 2017; Meng et al., 2021), while kaempferol and naringenin elevated the Cmax of lovastatin (Li et al., 2007).

**TABLE 2:** Statin-polyphenol interactions: Pharmacokinetic outcomes.

Statin [Dose]	Polyphenol [Dose]	Type of study	Pharmacokinetic parameters					References
			AUC	C_max_	T_max_	T_1/2_	CL/F	
Rosuvastatin [20 mg; day 1, 4 and 15, PO]	EGCG [300 mg/day; day 4–15; PO]	Clinical	↓	↓	N.D.	N.D.	N.D.	Kim et al. (2017)
Rosuvastatin [20 mg, SD, PO]	Baicalin [50 mg, 3 i.d., 14 days; PO]	Clinical	↓	=	=	↓	↑	Fan et al. (2008)
Rosuvastatin [50 mg/kg, SD, PO]	Naringin [42 mg/kg, SD, PO]	Non-clinical (*In vivo*)	=	=	=	=	=	Zeng et al. (2018)
Rosuvastatin [3 mg/mL/kg, SD, PO]	Phloretin [30 μg/mL/kg, SD, PO]	Non-clinical (*In vivo*)	=	N.D.	N.D.	N.D.	N.D.	Takahashi et al. (2021)
Rosuvastatin [10 mg, SD, PO]	Silymarin [140 mg, 3 i.d.; 5 days, PO]	Clinical	=	=	=	=	=	Deng et al. (2008)
Rosuvastatin [10 mg/kg, PO]	Scutellarin [50 mg/kg, PO]	Non-clinical (*In vivo*)	↑	↑	=	=	↓	Liu et al. (2020b)
Rosuvastatin [10 mg/kg, SD, PO]	Scutellarin [50 mg/kg, SD, PO]	Non-clinical (*In vivo*)	↑	↑	=	=	↓	Liu et al. (2020a)
Rosuvastatin [0.03 mg/kg, SD, PO]	Theaflavin, theaflavin-3-gallate, theaflavin-3′gallate; theaflavin-3,3-digallate [1.67 mL/kg, SD, PO]	Non-clinical (*In vivo*)	↓	↓	=	N.D.	N.D.	Kondo et al. (2019)
Simvastatin [40 mg/kg, SD, PO]	Baicalein [20 mg/kg, 10 days, PO]	Non-clinical (*In vivo*)	↑	↑	↓	↑	↓	Meng et al. (2021)
Simvastatin [40 mg/Kg, SD, PO]	Breviscapine [20 mg/Kg 8 days, IV]	Non-clinical (*In vivo*)	↑	↑	↑	↑	↓	Ju et al. (2017)
Simvastatin [20 mg/Kg, SD, IV]	EGCG [5 mg/kg, SD, IV]	Non-clinical (*In vivo*)	↑	N.D.	N.D.	↑	↓	Yang et al. (2017)
Simvastatin [0.25 mg/kg/d, 1 week]	Quercetin [10 mg/kg, 1 week]	Non-clinical (*In vivo*)	↓	↓	=	=	N.D.	Cermak et al. (2009)
Simvastatin metabolite [80 mg/kg, SD, PO]	Silymarin [45 mg/kg, SD, PO]	Non-clinical (*In vivo*)	↑	↑	=	=	N.D.	Li et al. (2019)
Atorvastatin [40 mg, SD, PO]	Green tea extract (Catechins, EGCG) [300 and 600 mg, SD, PO]	Clinical	↓	↓	=	=	↑	Abdelkawy et al. (2020)
Atorvastatin [20 mg/kg, SD, PO]	Quercetin [10 mg/kg, SD, PO]	Non-clinical (*In vivo*)	=	=	=	=	=	Koritala et al. (2015)
Atorvastatin [10 mg/kg, 7 days, PO]	Silymarin [50 mg/kg, SD, PO]	Non-clinical (*In vivo*)	↑	↑	↑	↑	↓	Malekinejad et al. (2014)
Lovastatin [10 mg/kg, SD, PO]	Kaempferol [2 and 10 mg/kg, SD, PO] Narigenin [2 and 10 mg/kg, SD, PO]	Non-clinical (*In vivo*)	↑	=	=	N.D.	N.D.	Li et al. (2007)
Fluvastatin [50 mM]	EGCG [0–100 µM]	Clinical	=	=	=	=	N.D.	Misaka et al. (2018)
Pravastatin [40 mg, SD, PO]	Quercetin [500 mg, 14 days, PO]	Clinical	↑	↑	=	↑	↓	Wu et al. (2012)

Abbreviations: AUC, area under the curve; Cmax, maximum plasma concentration; CL/F, clearance/fraction of dose available in the systemic circulation: clearance adjusted for bioavailability; EGCG, Epigallocatechin-3-gallate; T1/2, elimination half-life; Tmax, time to Cmax.; SD, single dose; PO, *per os*.

In contrast to the observed changes in AUC and Cmax, the time to reach maximum concentration (Tmax) remained unchanged in thirteen studies, suggesting that polyphenols have a modest impact on statins’ absorption rate. Regarding half-life (t1/2), most studies (nine) reported no reduction in the time required for statins to reach half of their initial concentration; five studies observed an increase, while only one noted a decrease. Similarly, clearance adjusted for bioavailability (CL/F), which reflects the efficiency of drug elimination after accounting for absorption, was reduced in most studies (seven), with two reporting increases and three finding no significant differences.

At the clinical level, only six studies addressed statin-polyphenol interactions, yielding conflicting results. For example, silymarin exhibited no effect on rosuvastatin pharmacokinetics (Deng et al., 2008), while EGCG and glycosylated baicalein decreased rosuvastatin total exposure, with glycosylated baicalein further increasing CL/F (Fan et al., 2008; Kim et al., 2017). EGCG also inhibited both the total exposure (AUC) and extent of exposure (Cmax) of atorvastatin while increasing its CL/F (Abdelkawy et al., 2020). Conversely, quercetin increased both the total exposure (AUC) and extent of exposure (Cmax) of pravastatin while reducing its CL/F (Wu et al., 2012). Unfortunately, no clinical studies have been conducted to investigate potential interactions between polyphenols and statins in their pro-drug forms. Moreover, aside from EGCG and scutellarin, for which studies have demonstrated both pharmacokinetic and pharmacodynamic interactions, very few experimental designs allow for the simultaneous evaluation of both effects.

## 4 Discussion

**FIGURE 6:**
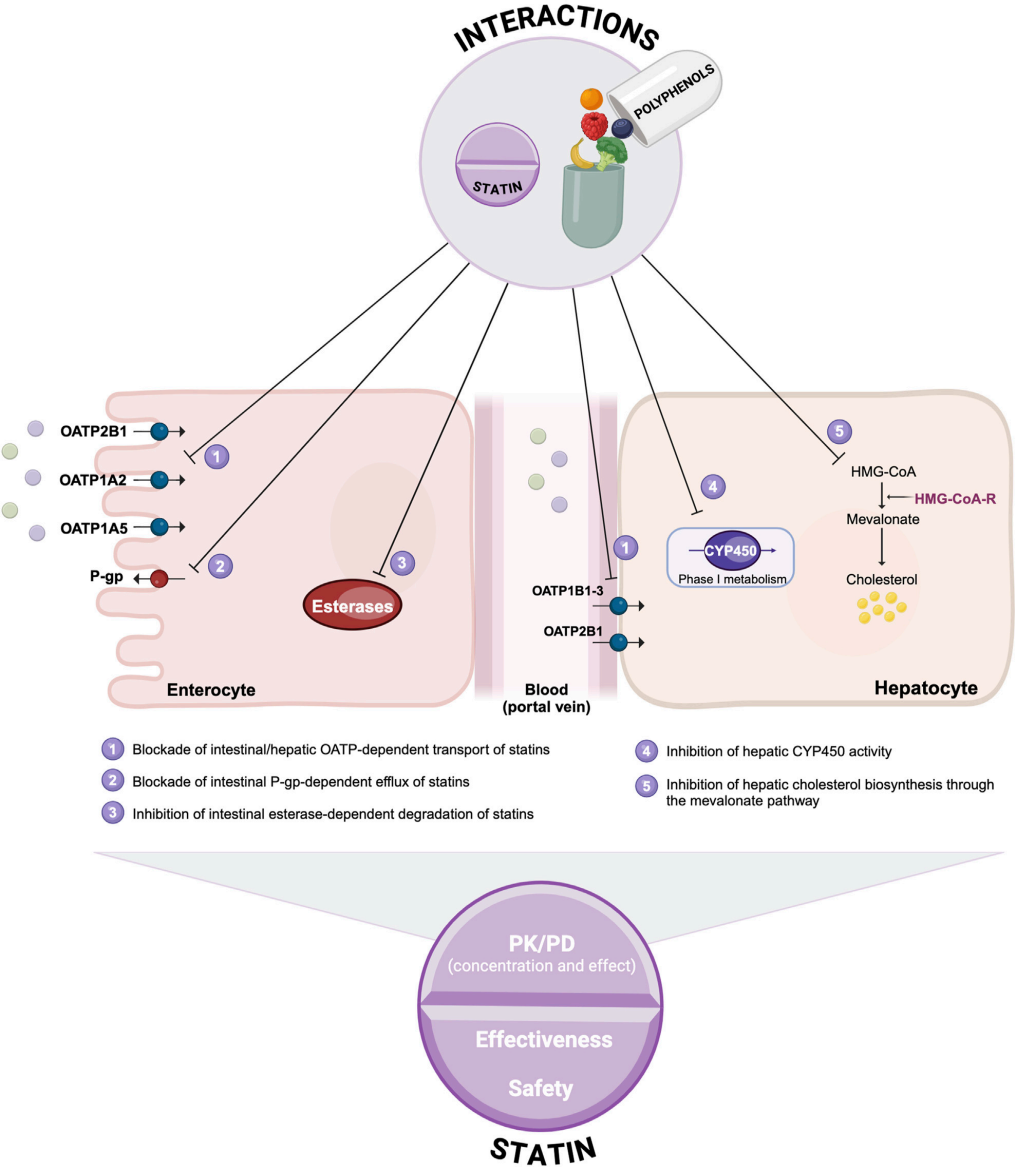
Putative mechanistic framework highlighting the biological mechanisms governing statin-polyphenol interactions (Image created in BioRender.com).

This comprehensive scoping review provides an integrated perspective on the cellular and molecular mechanisms underlying statin-polyphenol interactions, as summarized in Figure 6.

From a pharmacokinetic standpoint, polyphenols can modulate statin cellular uptake by inhibiting OATP and P-gp transporters. These effects may be particularly significant in the clinical practice for hydrophilic statins, whose gastrointestinal absorption and hepatic disposition rely heavily on carrier-mediated processes. Such inhibition can reduce overall statin exposure and, in some cases, may compromise drug efficacy. Given that polyphenols, through P-gp inhibition, are also expected to interfere with statin excretion into the bile, further studies are necessary to evaluate the impact of this potential mechanism on statin clearance.

Beyond their impact on cellular uptake, polyphenols also influence statin metabolism by inhibiting CYP450 enzymes, typically enhancing systemic statin exposure and consequently increasing the likelihood of adverse reactions and, ultimately, statin intolerance. Moreover, polyphenols can inhibit the esterase-mediated breakdown of statin prodrugs in the intestine, thereby enhancing the bioavailability of their active forms.

At a pharmacodynamic level, polyphenols (e.g., flavonoids) have been shown to inhibit HMG-CoA reductase at both the transcriptional level and enzyme activity. These interactions are synergistic, resulting in greater HMG-CoA reductase inhibition when statins and polyphenols are co-administered. Indeed, Scolaro and colleagues demonstrated that statin dose reductions may be feasible when complemented with polyphenol-based nutraceuticals in clinical practice (Scolaro et al., 2018). However, further studies are required to determine whether polyphenol supplementation may reduce or exacerbate statin-induced hepatotoxicity or myopathy, as inhibition of the mevalonate pathway appears to be a potential risk factor (Marcoff and Thompson, 2007; Abdel-Daim et al., 2018). Moreover, since polyphenols can inhibit dietary cholesterol uptake in enterocytes (Langhi et al., 2023), a deeper understanding of these interactions is essential to harness their synergistic effects as a potential strategy for reducing the burden of cardiovascular diseases (Gheorghe et al., 2020; Liao et al., 2024). Additionally, given that polyphenols can lower circulating cholesterol oxidation products and enhance statin efficacy (Ogino et al., 2007; Cilla et al., 2017; Coimbra et al., 2019), future research should explore whether they could also act as effective removal agents for exogenous toxic cholesterol oxidation products found in processed and fast foods, an area of considerable interest to the food and chemical industries.

The outcomes of statin-polyphenol interactions have demonstrated substantial inter-individual variability across studies, likely attributable to various methodological limitations. Notably, there is significant heterogeneity in the doses employed, both for statins (e.g., 0.03–10 mg/kg for rosuvastatin) and polyphenols (e.g., 20–50 mg/kg for baicalein), as well as in dosing regimens (e.g., single-dose protocols versus 28-day subacute protocols). Furthermore, the lack of standardized protocols is evident, with critical factors - such as the timing of administration and the concurrent consumption of food - often omitted, despite their known influence on the therapeutic response to statins.

Overall, this work presents an integrated perspective on the cellular and molecular mechanisms underlying statin–polyphenol interactions. It highlights conflicting evidence, from synergistic effects enabling lower statin doses and reduced adverse events to potential exacerbation of side effects. These findings underscore the need for controlled studies to clarify the clinical relevance of polyphenol-induced alterations in statin pharmacokinetics and pharmacodynamics. Such data are essential to develop evidence-based guidelines that may optimize statin therapy and support individualized treatment strategies. Future studies investigating the therapeutic effects of statins in individuals using polyphenol-based supplements and/or adhering to a polyphenol-enriched diet are warranted to optimize prescription practices and enhance statin’s effectiveness.

## Data Availability

The original contributions presented in the study are included in the article/supplementary material, further inquiries can be directed to the corresponding author.
